# Downregulation of lncRNA-11496 in the Brain Contributes to Microglia Apoptosis *via* Regulation of Mef2c in Chronic *T. gondii* Infection Mice

**DOI:** 10.3389/fnmol.2020.00077

**Published:** 2020-05-15

**Authors:** Xiahui Sun, Ting Wang, Yongliang Wang, Kang Ai, Ge Pan, Yan Li, Chunxue Zhou, Shenyi He, Hua Cong

**Affiliations:** ^1^Department of Pathogenic Biology, School of Basic Medical Sciences, Cheeloo College of Medicine, Shandong University, Jinan, China; ^2^College of Animal Science and Technology, Jilin Agricultural University, Changchun, China

**Keywords:** HDAC2, long non-coding RNA, Mef2c, microglia, *Toxoplasma gondii*

## Abstract

Though it is well known that chronic infections of *Toxoplasma gondii* (*T. gondii*) can induce mental and behavioral disorders in the host, little is known about the role of long non-coding RNAs (lncRNAs) in this pathological process. In this study, we employed an advanced lncRNAs and mRNAs integration chip (Affymetrix HTA 2.0) to detect the expression of both lncRNAs and mRNAs in *T. gondii* Chinese 1 strain infected mouse brain. As a result, for the first time, the downregulation of lncRNA-11496 (NONMMUGO11496) was identified as the responsible factor for this pathological process. We showed that dysregulation of lncRNA-11496 affected proliferation, differentiation and apoptosis of mouse microglia. Furthermore, we proved that Mef2c (Myocyte-specific enhancer factor 2C), a member of the MEF2 subfamily, is the target gene of lncRNA-11496. In a more detailed study, we confirmed that lncRNA-11496 positively regulated the expression of Mef2c by binding to histone deacetylase 2 (HDAC2). Importantly, Mef2c itself could coordinate neuronal differentiation, survival, as well as synapse formation. Thus, our current study provides the first evidence in terms of the modulatory action of lncRNAs in chronic toxoplasmosis in *T. gondii* infected mouse brain, providing a solid scientific basis for using lncRNA-11496 as a therapeutic target to treat *T. gondii* induced neurological disorder.

## Introduction

*Toxoplasma gondii* (*T. gondii*) is a protozoan that is widely parasitic in nucleated cells of humans and animals. According to a serological survey, about 30% of the world’s population is infected with this parasitic protozoan. As an opportunistic pathogenic parasite, *T. gondii* can cause severe toxoplasmosis only in an immunocompromised host. In a host with normal immune function, it usually manifests as an asymptomatic latent infection (Wohlfert et al., 2017). However, in recent years, an increasing number of studies have found that a *T. gondii* latent infection is not asymptomatic, but rather results in intellectual changes, behavioral abnormalities and even mental illness in its host (Hamdani et al., 2017; Tyebji et al., 2019).

A growing body of evidence indicated that a chronic *Toxoplasma* infection can cause mental disorders. For instance, *T. gondii* infections are positively correlated with the occurrence of depression and schizophrenia (Tedford and McConkey, 2017; Xiao et al., 2018). The relationship between Toxoplasma infection and epilepsy as well as neurodegenerative diseases, such as Parkinson’s and Alzheimer’s disease, has also attracted extensive attention from researchers (Ngô et al., 2017). *T. gondii* causes damage to the central nervous system of the host which might result in abnormalities in the mental state and behavior of the host no matter it is a congenital or acquired infection (Khan and Khan, 2018; Martinez et al., 2018). Therefore, it is necessary to explore the molecular mechanism of brain damage caused by *Toxoplasma* infection to find a strategy for early prevention and treatment.

It has been discovered that *T. gondii* tachyzoites invade monocytes and dendritic cells through the blood-brain barrier in a “trojan horse” manner, and gradually transforms into a neutrophil to form cysts during the host’s immune response (Mendez and Koshy, 2017). Previous studies have shown that *T. gondii* cysts have certain selectivity for different regions of mouse brain tissue, but the relationship between the cyst’s location and host’s mental and behavioral changes remains inconclusive (Blanchard et al., 2015). *T. gondii* has a high degree of neurotropic action, which can actively invade the host’s nerve cells, causing direct and indirect damage to nerve cells (Cabral et al., 2016). Activation of microglia and astrocytes protects the central nervous system, but persistently secreted cytokines activate the inflammatory pathway which triggers excessive immune responses, leading to neuronal apoptosis and neurotransmitters abnormal secretion (Wang et al., 2019). However, the exact regulatory molecules that play a key role in the process of *T. gondii* infection in the brain are unknown.

In recent years, with the development of high-throughput sequencing technology, transcriptomics has become a new direction to discover the mechanism of pathogens (Hakimi et al., 2017). Recent studies have found that thousands of long non-coding RNAs (lncRNAs) with a length of more than 200 nucleotides, conventionally defined as non-translated RNA, were found to play important regulatory roles in transcriptional regulation and epigenetic processes (Andersen and Lim, 2018). It has been known that lncRNAs are preferentially expressed in the nervous system in highly precise Spatio-temporal patterns, and several of these lncRNAs are found to play an important role in the regulation of brain evolution, development and synaptic plasticity (Atianand et al., 2016; Kleaveland et al., 2018). However, little is known about the modulatory action of lncRNAs in chronic toxoplasmosis.

Because the dominant *T. gondii* genotype Chinese 1 wh6 strain in China has effector molecules and host immune response mechanisms that are different from the prototype strain, it is of great significance to explore the expression pattern and function of lncRNAs in the brain of mice with chronic infection of this genotype strain. In this study, lncRNAs and mRNAs integration chip (Affymetrix HTA 2.0) was set up to detect the expression of lncRNAs and mRNAs in the brains of mice infected with the *T. gondii* Chinese 1 strain. We found that the expression of lncRNAs in the brain of mice infected with *T. gondii* varied greatly when comparing to that of the uninfected mice. Among them, the down-regulation of lncRNA-11496 (NONMMUGO11496) was first identified in the brain of *T. gondii* infected mice. The role of lncRNA-11496 and its underlying mechanism in the brain of mice chronically infected with *T. gondii* was further explored. This was the first study to uncover the mechanism of modulatory action of lncRNAs in chronic toxoplasmosis induced by *T. gondii* Chinese 1 strain.

## Materials and Methods

### Ethical Approval

All animal experimental procedures used in the present study had been given prior approval by the Institutional Animal Care and Use Committee of Shandong University under Contract LL201602044. Humane endpoints were chosen to terminate the pain or distress of experimental animals *via* euthanasia. Mice were monitored daily for 8 weeks for signs of toxoplasmosis, which includes: food and water intake difficulties, fatigue, and severe ascites. Any mouse that showed signs of illness was euthanized with carbon dioxide.

### Mice

Thirty specific-pathogen-free female BALB/c mice (6–8 weeks old) were delivered from Shandong University Laboratory Animal Centre (Jinan, China). The mice were housed five per cage under pathogen-free conditions and were adequately supplied with sterilized water and food.

### Parasite and Infection

*T. gondii* Chinese 1 genotype, wh 6 (TgCtwh6) strain, a low virulent strain and usually cause chronic infection and shaped tissue-cysts, which was the main clonal lineage in China (Li et al., 2014), was a gift from Ji Long Shen, professor of Anhui Medical University. A chronic *T. gondii* infected BALB/c mice model was established *via* administration by gavage at a dose of 20–30 cysts. All experiments in the present study were performed 8 weeks after infection.

### Microarray Assay

lncRNAs and mRNAs microarray assay were performed by the Biotechnology Corporation (Shanghai, China). Total RNA was extracted from the brains of mice using TAKARA RNAiso by following the manufacturer’s instructions. RNA was checked for a RIN number to inspect RNA integrity by an Agilent Bioanalyzer 2100 (Agilent Technologies, Santa Clara, CA, USA). Qualified total RNA was further purified by RNeasy mini kit (QIAGEN, Hilden, Germany) and RNase-Free DNase Set (QIAGEN, Hilden, Germany). Total RNA was amplified and labeled by Low Input Quick Amp WT Labeling Kit (Agilent Technologies, Santa Clara, CA, USA). Labeled cRNA was purified by RNeasy mini kit (QIAGEN, Hilden, Germany). Each slide was hybridized with 1.65 μg Cy3-labeled cRNA using Gene Expression Hybridization Kit (Agilent Technologies, Santa Clara, CA, USA) in Hybridization Oven (Agilent Technologies, Santa Clara, CA, USA). After 17 h of hybridization, the slides were washed in staining dishes (Thermo Fisher Scientific, Waltham, MA, USA) with Gene Expression Wash Buffer Kit (Agilent Technologies, Santa Clara, CA, USA) according to the manufacturer’s instructions. Slides were scanned by Agilent Microarray Scanner (Agilent Technologies, Santa Clara, CA, USA) with default settings, Dye channel: Green, Scan resolution = 3 μm, PMT 100%, 20 bit. Data were extracted with Feature Extraction software 10.7 (Agilent Technologies, Santa Clara, CA, USA) and normalized by Quantile algorithm, limma packages in R.

### Gene Ontology (GO) and Kyoto Encyclopedia of Gene and Genomes (KEGG) Pathway Analyses

To understand the function of differentially expressed genes in the microarray profiles, we performed Gene Ontology (GO) and Kyoto Encyclopedia of Gene and Genomes (KEGG) pathway analysis for Annotation, Visualization and Integrated Discovery (DAVID v6.7). The dysregulated lncRNAs and differentially expressed mRNA fold change ≥2, *P*-value < 0.05 were selected.

### Prediction of the Target Genes of lncRNA

Differentially expressed lncRNAs (Fold change ≥2) were selected for potential target-gene prediction *via* cis or trans-regulatory. Two independent algorithms were used. The first algorithm searches for target genes acting in cis. Using gene annotations at UCSC^1^, lncRNAs and potential target genes were paired and visualized using the UCSC genome browser. The genes transcribed within 10 kb region upstream or downstream of lncRNAs were considered as cis or trans target genes.

### RNA-FISH

Fluorescence-conjugated lncRNA-11496 probes were used for fluorescence *in situ* hybridization (FISH) analysis. Routine RNA-FISH testing was performed as previously described (Yu et al., 2018). With known nucleotide probes, the target RNA was formed according to the principle of pairing with lncRNA-11496 complementary bases. In strict accordance with Genepharma’s procedures, the location of the target RNA can be directly observed by Zeiss fluorescence confocal microscopy or MIDI panoramic scanning of nucleic acid probe hybridization of cells and tissues under qualitative and quantitative or relatively localized target RNA.

### Cell Culture

BV-2 cell, mouse microglia cell, gifted to us from Dr. Yan Zhang, Shandong University, and was maintained in our lab. BV-2 cells were cultured in Dulbecco’s modified Eagle’s medium (DMEM, HyClone, USA) containing 10% fetal bovine serum and 1% Penicillin-Streptomycin solution (Beyotime, China) at 37°C in an incubator containing 5% CO_2_.

### Plasmids Construction

pGPU6/GFP/Neo vector expressing the short hairpin RNAs (shRNA) was constructed to silence lncRNA-11496. Sequence of shlncRNA-11496 was shown as follows: 5′CACCGCTCAATGT ATCCAGTTTAACTTCAAGAGAGTTAAACTGGATACATTG AGCTTTTTTG-3′. oelncRNA-11496 was constructed with pCDNA3.1(+) vector used to overexpress lncRNA-11496.

### Quantitative Real-Time PCR (qRT-PCR) Assay

Total RNA from mouse brains and cell lines was extracted using EASYspin Plus Tissue/Cell RNA Rapid Extraction Kit according to the manufacturer’s instructions (Aidlab, China). The quantity of RNA was determined by spectrophotometry and electrophoresis. The total RNA was reversely-transcribed using HiScript^®^II QRT SuperMix for qPCR)(+gDNA wiper) Kit (Vazyme, China). Quantitative real-time PCR (qRT-PCR) was performed using ChamQ^TM^ SYBR^®^ qPCR Master Mix (Vazyme, China). GAPDH was used as an internal control. qRT-PCR was performed using LightCycler^®^96 (Roche, Switzerland). And the relative expression of the target RNA was calculated using the 2^−ΔΔCt^ method. The primers sequences were listed in Supplementary Table S1. All experiments were performed at least three times to ensure accuracy.

### Western Blot Analysis

Total proteins were prepared by RIPA Lysis Buffer (Beyotime, China) containing phenylmethanesulfonylfluoride (PMSF). Protein concentration was determined using a BCA protein assay kit (Beyotime, China). Briefly, the proteins were separated on Sodium Dodecyl Sulfate Polyacrylamide gel electrophoresis (SDS-PAGE) and transferred onto polyvinylidene difluoride (PVDF) membrane (Millipore, Kankakee, IL, USA). The membrane was blocked in 5% Albumin Bovine V (BCA) for 1 h. The PVDF membrane was incubated with primary antibodies, including rabbit anti-MEF2C antibody (1:5,000, Abcam, Cambridge, MA, USA), rabbit anti-HDAC2antibody (1:1,000, Abcam, Cambridge, MA, USA) and rabbit anti-GAPDH antibody (1:5,000, Biosynthesis, China), overnight at 4°C. Following extensive washing with TBST, secondary antibodies (Goat Anti-Rabbit IgG H&L, 1:2,000, Abcam, Cambridge, MA, USA) were incubated at room temperature for 1 h. The labeled proteins were measured using UltraECL Chemiluminescence Kit (Aidlab, China).

### Immunohistochemistry and Immunofluorescence Assays

Immunohistochemical staining was performed according to the manufacturer’s protocol using the DAB reagent (Servicebio, China). Brain slides derived from infected mice were deparaffinized and rehydrated using xylene and ethanol; and then, the slides were immersed in Sodium citrate antigen retrieval solution (pH 6.0) and 3% H_2_O_2_ and then were incubated at room temperature to block endogenous peroxidase (Block with 3% BSA at room temperature). Rabbit anti-MEF2C antibody (1:500, Abcam, Cambridge, MA, USA) and rabbit anti-HDAC_2_ antibody (1:500, Servicebio, China) were used as primary antibodies. The secondary antibody was labeled with HRP Goat Anti-Rabbit IgG and nucleus stained with Hematoxylin staining solution.

For the immunofluorescence assay, the sections were incubated with CY3-conjugated goat anti-rabbit IgG(H+L; 1:300, Servicebio, China) at 37°C for 1 h after incubating with the primary antibodies overnight at 4°C. Then, the samples were stained with 4′6-diamidino-2-phenylindole (DAPI; Servicebio, China) and then were examined under a fluorescence microscope (Nikon Eclipse C1, Japan).

### Cells Transfection

When the cells grew to 70–80% confluence in 6-well culture plates, the cells were transfected with a plasmid (shlncRNA-11496 and oelncRNA-11496) using Micropoly-transfecter^TM^ Cell Reagents (Micropoly, China) according to the manufacturer’s instructions. The efficiency of silence or overexpression of lncRNA-11496 was measured by qRT-PCR 48 h after transfection.

### Cell Counting Kit-8 Assay

To evaluate the proliferation of BV-2 cells, the cells were counted by Cell Counting Kit (CCK-8, Dojindo, Japan). Briefly, the cells were seeded into 96-well plates at a density of 1 × 10^4^ cells per well in 100 μl medium; and then cells were transfected with shRNA and overexpression plasmid by Micropoly-transfecter^TM^ Cell Reagent. Subsequently, 10 μl of CCK-8 reagent was added to each well and incubated at 37°C for 1–2 h. The absorbance of optical density was determined using the Automatic Enzyme Label Analyzer (Allsheng, China) at 450 nm (A450). Each experiment was performed in triplicate and repeated three times to ensure accuracy.

### Cell Colony Formation Assays

To assess the proliferation of BV-2 cells, we performed a cell colony formation experiment. Three-hundred cells transfected with shRNA plasmid, overexpression plasmid or control plasmid were seeded in 6-well plates and cultured in 37°C, 5% CO_2_ conditions. Two weeks later, the plates were washed with phosphate-buffered saline (PBS), and stained with crystal violet for 10 min. Then the plates were photographed and colony numbers were counted. All experiments were performed in triplicate to ensure accuracy.

### TUNEL Assay

BV-2 cells were seeded on coverslips in 6-well plates and were transfected with shRNA and overexpression plasmid *via* Micropoly-transfecter^TM^ Cell Reagent. After 48 h, cells were fixed in paraformaldehyde for 20 min at room temperature. Cell apoptosis was detected using TUNEL assay kit (Beyotime, China) according to the manufacturer’s instructions. Cell nuclei were incubated with DAPI solution. The coverslips were observed and images are collected by Fluorescent Microscopy. Apoptosis cells are labeled with FITC which show green fluorescence.

### Cell Cycle Analyses

BV-2 cells were collected by centrifugation after cell transfection. Pre-cooling (−20°C) 75% ethanol was added to fix cells overnight at 4°C. The cells were washed with a PBS solution and were removed RNA using RNase and incubated with PI solution at 37°C for 40 min. The cells labeled by fluorescent are detected by Flow Cytometer. The cells cycles were analyzed with Modfit software.

### RNA Pull-Down and Mass Spectrometry Assay

lncRNA11496 and its antisense RNA were transcribed from vector pCDNA3.1(+) by using the MEGAscript^®^ Kit (Thermo Fisher Scientific, Waltham, MA, USA). lncRNAs and antisense RNAs were labeled using magnetic beads and were incubated with 20–200 μg of BV-2 cell protein. After 1 h incubation, the proteins were washed with Wash Buffer using Pierce^TM^ Magnetic RNA-Protein Pull-down Kit according to the manufacturer’s instructions (Thermo Fisher Scientific, Waltham, MA, USA). The associated proteins were detected by Mass Spectrometry and Western blot.

### Statistical Analysis

The results are expressed as the mean ± standard error of the mean (SEM). Data were analyzed with a two-tailed student’s *t*-test using GraphPad Prism 7. *P* < 0.05 and was considered statistically significant.

## Results

### Differential Expressions of lncRNAs-mRNAs Were Analyzed in the Brain of Mice Infected With *T. gondii*

**Figure 1:**
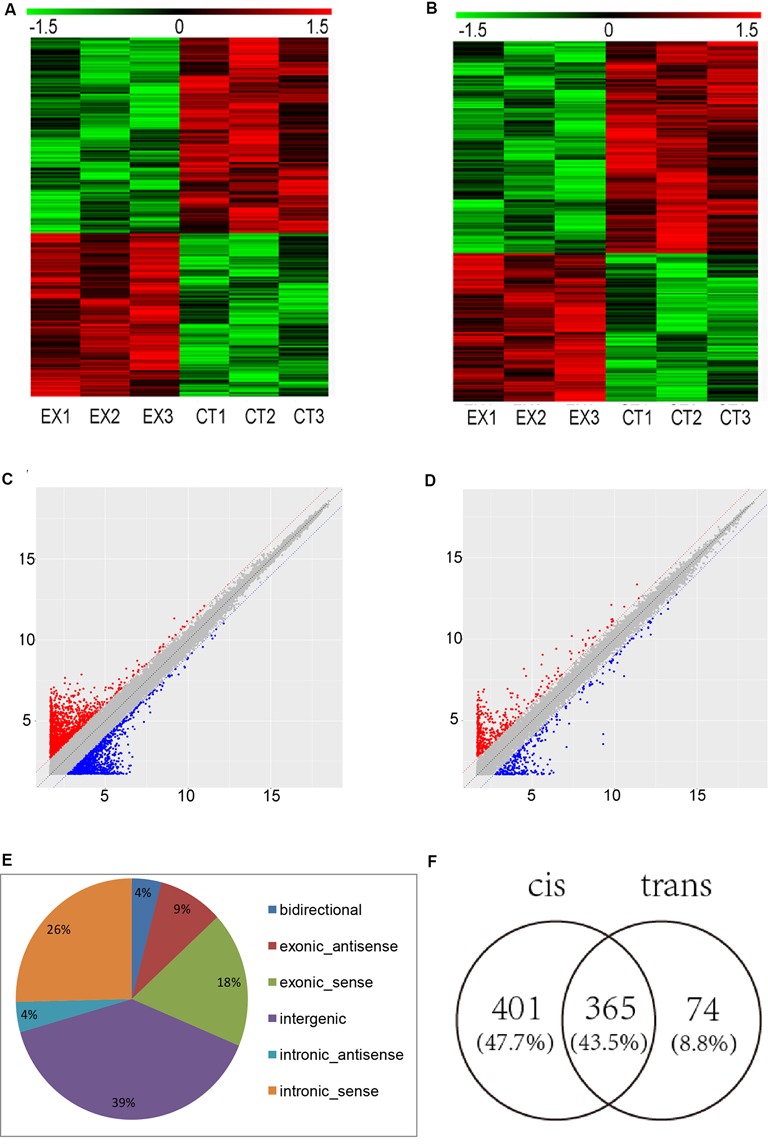
Microarray analysis for long non-coding RNAs (lncRNAs) and mRNAs expression in the brains of mice infected with *T. gondii* Chinese1 Wh 6 strain.** (A)** Hierarchical clustering of differentially expressed lncRNAs in the brain of mice infected and uninfected with *T. gondii*. Values in the color scale are normalized intensities. Red bands indicate high relative expression and green bands indicate low relative expression. **(B)** Hierarchical clustering of differentially expressed mRNAs in the brain of mice infected and uninfected with *T. gondii*. Values in the color scale are normalized intensities. Red bands indicate high relative expression and green bands indicate low relative expression. **(C)** Volcano Scatter plot filtering was used to visualize fold regulation and statistical significance in lncRNA populations in mice brains. Statistically significant (*p* ≤ 0.05) up (red) or down (blue) regulated expression changes (>2-fold) are shown in pair-wise comparisons of *T. gondii* infected and uninfected mice brains. **(D)** Volcano Scatter plot filtering was used to visualize fold regulation and statistical significance in mRNAs populations in mice brains. Statistically significant (*p*-value ≤ 0.05) up (red) or down (blue) regulated expression changes (>2-fold) are shown in pair-wise comparisons of *T. gondii* infected and uninfected mice brains. **(E)** Classification of dysregulated lncRNAs in the brain of mice infected by *T. gondii*. **(F)** Venn diagram of the target genes of dysregulated lncRNAs using cis and trans analysis.

To determine the lncRNAs and mRNAs expression profiles in the brain of mice chronically infected and uninfected with *T. gondii* Chinese 1 Wh 6 strain, three matching pairs of brains from mice were collected for microarray analysis. Significant differential expression profiles for the infected and uninfected mice are shown in the heat map of hierarchical clustering (Figures 1A,B). The scatter plot map indicated the distribution and expression variation of the log 2 ratios of lncRNAs and mRNAs (Figures 1C,D). The result suggested that, compared with uninfected mice, 1,500 lncRNAs and 864 mRNAs were differentially expressed (*p* ≤ 0.05) in the brain of *T. gondii* infected mice, of which 662 lncRNAs and 356 mRNAs were up-regulated, while 838 lncRNAs and 508 mRNAs were down-regulated.

The differentially expressed lncRNAs were further classified into five categories based on their genomic locations: bidirectional, exonic-antisense, exonic-sense, intergenic, and intronic locations. They accounted for 4%, 9%, 18%, 39% and 30%, respectively. Interestingly, among all five categories of lncRNAs detected in this study, the intergenic lncRNAs were mostly altered in the brain of mice chronically infected with *T. gondii* (Figure 1E).

To understand whether dysregulated lncRNAs are involved in the regulation of genes and their associated signaling pathways in relevance to *T. gondii* latent infection of the brain, we predicted potential target genes of lncRNAs in the database using target prediction programs. Overall, 840 dysregulated lncRNAs were identified both in *cis* and *trans* target genes. Among them, 401 lncRNAs can predict target genes by *cis*, 74 by *trans*, and 365 by both *cis* and *trans* (Figure 1F). The target genes of differentially expressed lncRNAs were predicted and analyzed for further screening.

### KEGG and GO Enrichment Analysis of Differentially Expressed Genes

To find a biological regulatory pathway for significant differences in experimental conditions, we further classified the differential genes by KEGG and GO enrichment.

**Figure 2:**
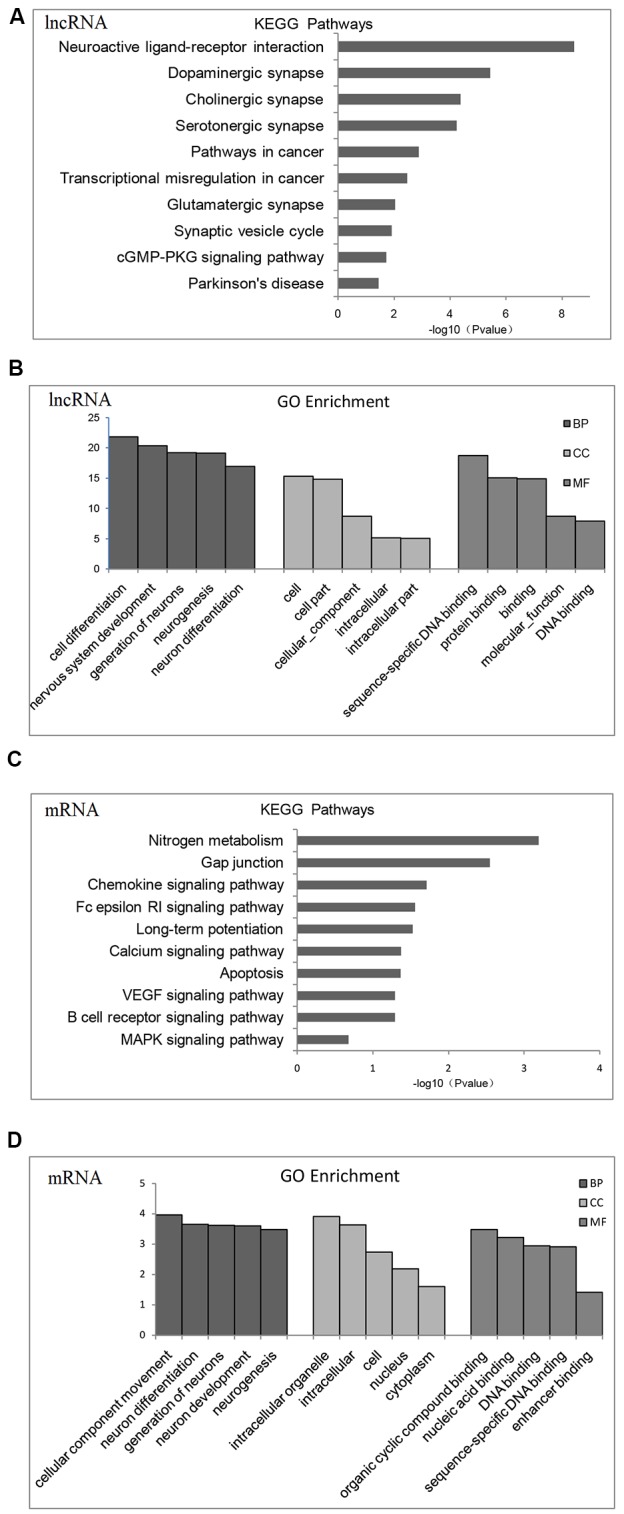
Gene Ontology (GO) functional enrichment and Kyoto Encyclopedia of Gene and Genomes (KEGG) pathways analysis of differentially expressed mRNAs and lncRNAs in the brains of chronic *T. gondii* infected mice. **(A)** KEGG pathways analysis for differentially expressed dysregulated lncRNAs target genes. The vertical axis represents the pathway category and the horizontal axis represents the enrichment score [−log_10_ (*p*-value)] of the pathway. **(B)** GO functional enrichment analysis for dysregulated lncRNAs target genes. The top five groups in the three main categories: biological process (BP), cellular component (CC) and molecular function (MF) are summarized. The *x*-axis represents the subcategories and the *y*-axis represents the enrichment scores [−log_10_ (*p*-value)] of the significant enrichment GO terms. **(C)** KEGG pathways analysis for differentially expressed dysregulated mRNAs. The vertical axis represents the pathway category and the horizontal axis represents the enrichment score [−log_10_ (*p*-value)] of the pathway. **(D)** GO functional enrichment analysis for dysregulated mRNAs. The top five groups in the three main categories: BP, CC and MF are summarized. The *x*-axis represents the subcategoriesand the *y*-axis represents the enrichment scores [−log_10_ (*p*-value)] of the significant enrichment GO terms.

According to KEGG pathway and GO enrichment, differential expression of lncRNA target genes is mostly enriched in neuroactive ligand-receptor interaction or dopaminergic, cholinergic, serotonergic, and glutamatergic synapse pathways that are related to proliferation and differentiation of nerve cells, apoptosis, cellular components (CCs), and DNA-binding (Figures 2A,B). In contrast, differentially expressed mRNA is mostly enriched in nitrogen metabolism, chemokines, VEGF signaling pathway, calcium signaling pathway, MAPK signaling pathway, differentiation and proliferation of neural cells, nucleic acid synthesis, and other related pathways (Figures 2C,D).

Fifteen categories of genes in biological process (BP), CC and molecular function (MF) are shown in GO enrichment. According to GO enrichment, the differentially expressed genes are mostly enriched in neuron system development, neuron differentiation, neuron development, neuron parthenogenesis synapse, et cetera (Supplementary Figure S1A). Furthermore, significant signaling pathways are shown in KEGG enrichment. We found that differentially expressed genes are mostly enriched in neuroactive ligand-receptor interactions, dopamine and glutamate synapses, and Parkinson’s disease (Supplementary Figure S1B).

### Validation of lncRNA-11496 Down-Regulation in Chronic *T. gondii* Infected Mouse Brain

**Figure 3:**
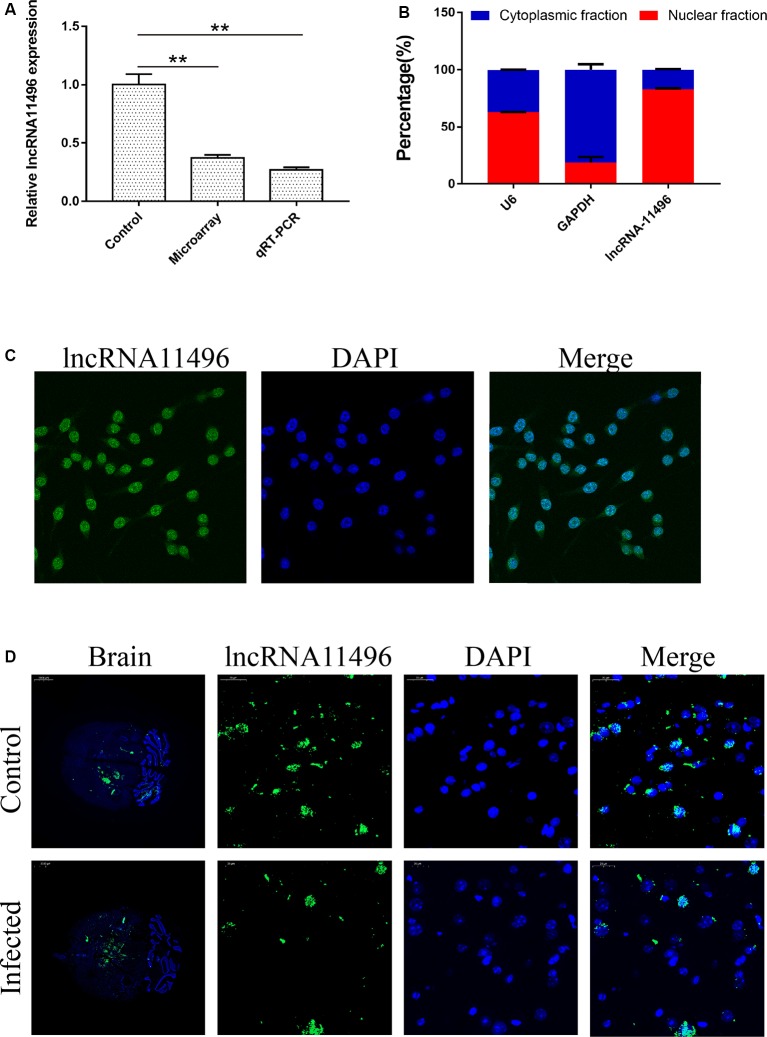
Validation of lncRNA-11496 down-regulation in chronic *T. gondii* infected mouse brain.** (A)** Validation of the expression of lncRNA-11496 by microassay and quantitative real-time PCR (qRT-PCR). All values are presented as mean ± SD for three independent experiments. **P < 0.01, student’s *t*-test. **(B)** A majority of lncRNA-11496 was expressed in the nucleus of BV-2 cells through cellular fraction assays. U6 served as a positive control for nuclear gene expression and GAPDH served as a positive control for cytoplasmic gene expression. **(C)** Fluorescence *in situ* hybridization (FISH) analysis demonstrated that lncRNA-11496 was distributed mostly in the nucleus of BV-2 cells. **(D)** FISH binding affinity of lncRNA11496 shows a significant down regulation trend in the brain of *T. gondii* infected mice compared with that of the uninfected mice.

LncRNAs with significant differences in expression were screened based on the GO and KEGG enrichment analysis for the differential expression of mRNAs and lncRNAs (Supplementary Figure S1C). Most significantly, down-regulated lncRNA NONMMUG011496 (lncRNA-11496) was identified in the brains of mice infected with *T. gondii* and further validated by Microassay and qRT-PCR (Figure 3A). As indicated in Figure 3B, the expression of lncRNA-11496 was higher in the nucleus than that in the cytoplasma of BV-2 cells by cellular fraction assays. The FISH analysis further confirmed that lncRNA-11496 is distributed mostly in the nucleus of BV-2 cells (Figure 3C). We further examined the expression pattern of lncRNA-11496 in the brains of mice infected with *T. gondii* Chinese 1strain. The FISH binding affinity analysis showed a significant downregulation of lncRNA-11496 in the brain of *T. gondii* infected mice compared with that of the uninfected mice (Figure 3D).

### The Prediction and Identification of the Target Gene of lncRNA-11496

**Figure 4:**
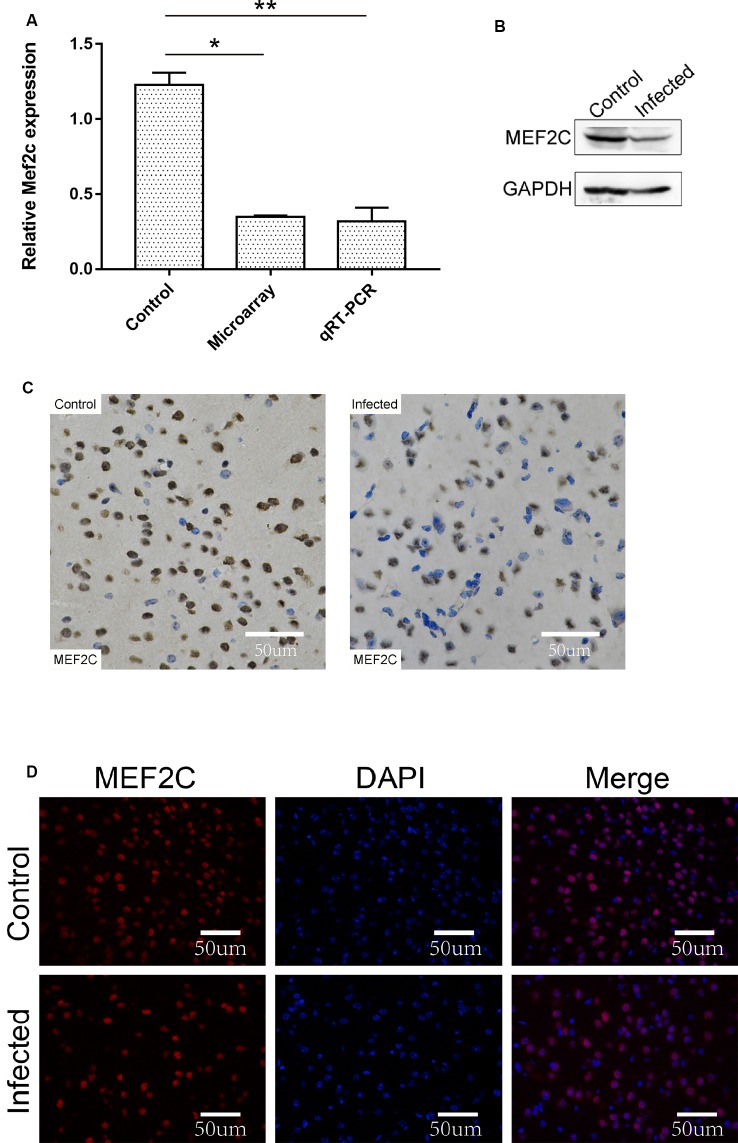
Validation of the expression of Mef2c in the brain of mice infected with *T. gondii*. **(A)** The expression of Mef2c in the brain of mice infected with *T. gondii* was tested by qRT-PCR. All values are presented as mean ± SD for three independent experiments (*P < 0.05, **P < 0.01, student’s *t*-test). **(B)** The expression of MEF2C protein in *T. gondii* infected and uninfected mouse brain was analyzed by Western blot. **(C)** Immunohistochemical analysis of MEF2C protein in *T. gondii* infected and uninfected mouse brain. The slice derived from brain was subsequently subjected to rabbit anti-MEF2C antibody and HRP Goat Anti-Rabbit IgG, and then nucleus stained with hematoxylin staining solution. MEF2C protein shows in yellow color. **(D)** Immunofluorescence analysis of MEF2C protein expression in *T. gondii* infected and uninfected mouse brain. The slice derived from brain was subjected to immunofluorescence with the primary antibody against MEF2C (red). DAPI (blue) was used for counterstaining.

The chromosome location of lncRNA-11496 was confirmed through the UCSC Genome Browser. lncRNA-11496 is located on mouse chromosome 13:83573618-83578409 and its transcript length is 3045bp (Supplementary Figure S2A). Through the analysis of target genes by *cis and trans*, lncRNA-11496 was identified to locate in the promoter region of Mef2c (Supplementary Figure S2B), indicating that Mef2c could be a potential target gene of lncRNA-11496. As shown in Figures 4A,B, the expression of the Mef2c gene and MEF2C protein in the brains of mice infected with *T. gondii* exhibited a significant decrease as compared to the uninfected group. Immunohistochemistry and immunofluorescence experiments also demonstrated that the expression of MEF2C in the brains of mice infected with *T. gondii* was significantly reduced compared to that of the uninfected mice (Figures 4C,D).

### lncRNA-11496 Positively Regulates the Expression of Mef2c in BV-2 Cells

**Figure 5:**
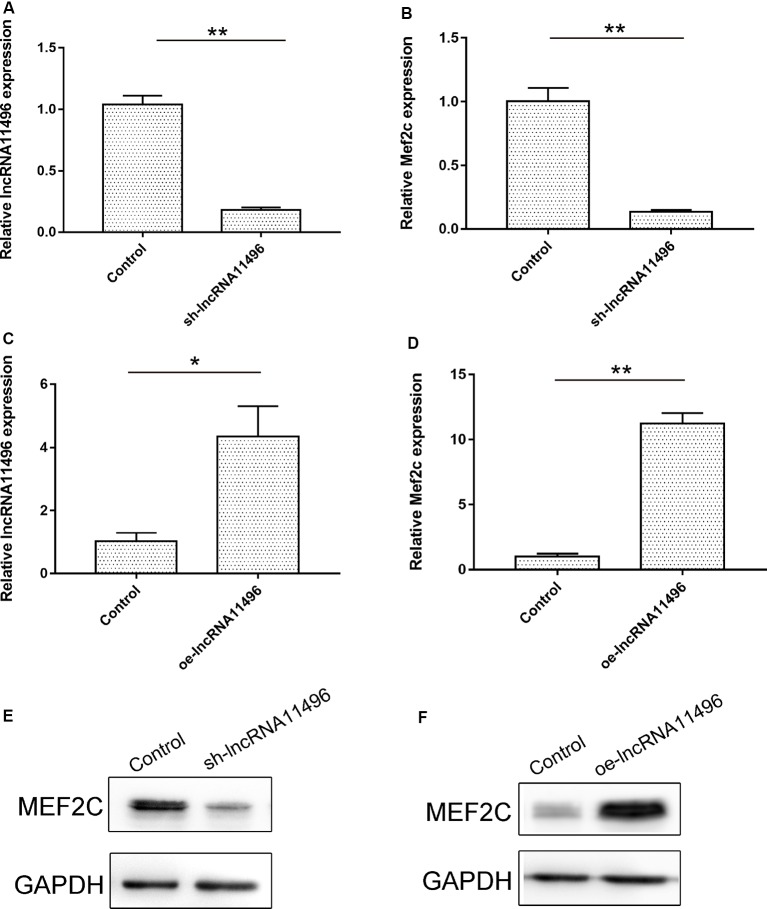
lncRNA11496 positively regulates the expression of Mef2c.** (A)** The expression of lncRNA11496 in BV-2 cells transfected with shlncRNA-11496 was tested by qRT-PCR. All values are presented as mean ± SD for three independent experiments (**P < 0.01, student’s *t*-test). **(B)** The expression of Mef2c in BV-2 cells transfected with shlncRNA-11496 was tested by qRT-PCR. All values are presented as mean ± SD for three independent experiments (**P < 0.01, student’s *t*-test). **(C)** The expression of lncRNA11496 in BV-2 cells transfected with oe-lncRNA11496 was tested by qRT-PCR. All values are presented as mean ± SD for three independent experiments (*P < 0.05, student’s *t*-test). **(D)** The expression of Mef2c in BV-2 cells transfected with oe-lncRNA11496 was tested by qRT-PCR. All values are presented as mean ± SD for three independent experiments (**P < 0.01, student’s *t*-test). **(E)** The expression level of MEF2C protein was decreased in BV-2 cells transfected with shlncRNA-11496 was tested by Western-blot. GAPDH was used as an internal control. **(F)** The expression level of MEF2C protein was enhanced in BV-2 cells transfected with oe-lncRNA11496 was tested by Western-blot. GAPDH was used as an internal reference control.

To verify if lncRNA-11496 could regulate the expression of Mef2c, sh-lncRNA11496 (lncRNA-11496 interfering plasmid) and oe-lncRNA11496 (lncRNA-11496 over-expression plasmid)were transfected into BV-2 cells to detect the expressions of lncRNA-11496 and Mef2c by qPCR and WB. The results showed that the expression of Mef2c was down-regulated when lncRNA11496 was knocked down (Figures 5A,B), while opposite results were observed when lncRNA-11496 was overexpressed (Figures 5C,D). This result was further confirmed using the lncRNA-11496 interference approach (Figures 5E,F). Thus, our results demonstrated that lncRNA-11496 can positively regulate the expression of Mef2c.

### Exploration of the Role of lncRNA-11496 in the Cell Cycle, Proliferation and Apoptosis in BV-2 Cells

To clarify the function of lncRNA-11496 in the neuron, sh-lncRNA11496 (lncRNA-11496 interfering plasmid) and oe-lncRNA11496 (lncRNA-11496 over-expression plasmid) were constructed to investigate the role of lncRNA-11496 in the cell cycle, proliferation and apoptosis in BV-2 cells.

**Figure 6:**
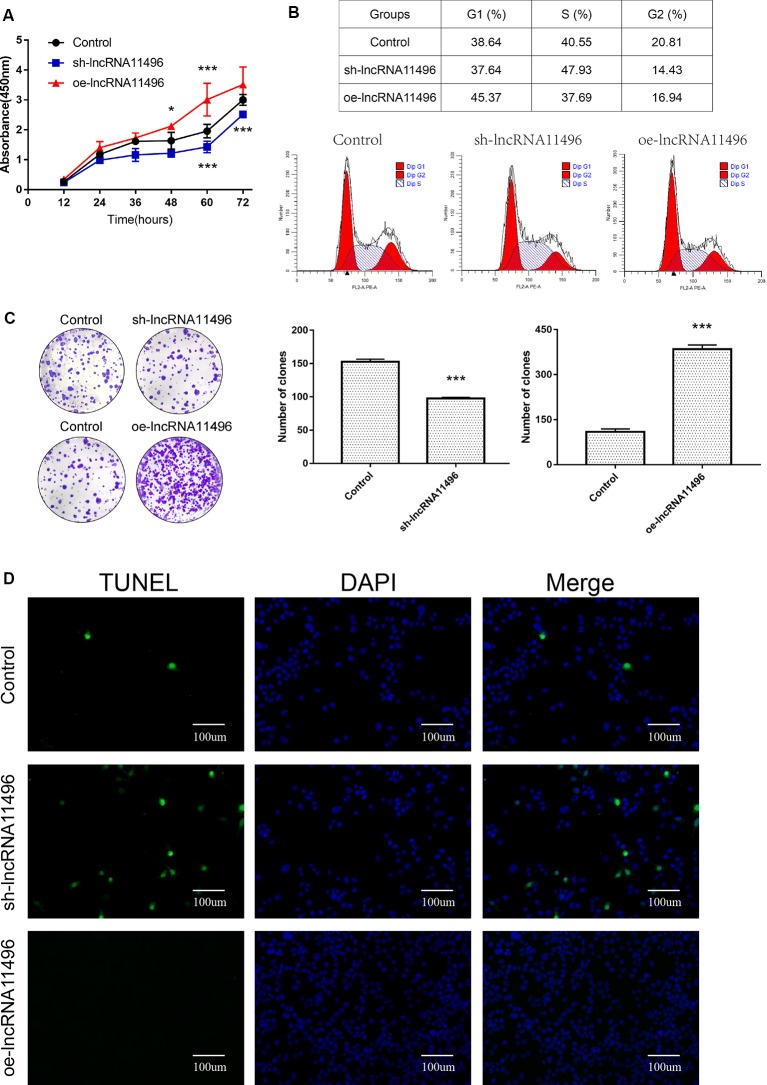
lncRNA11496 affects proliferation, differentiation and apoptosis of BV-2 cells.** (A)** Cell proliferation was determined by CCK-8 assay which was performed in BV-2 cells transfected with sh-lncRNA11496 or oe-lncRNA11496 12 h, 24 h, 36 h, 48 h, 60 h, 72 h later. All values are presented as mean ± SD for three independent experiments (*P < 0.05, ***P < 0.001, student’s *t*-test). **(B)** The cell cycle of BV-2 cells transfected with sh-lncRNA11496 or oe-lncRNA11496 was analyzed by Flow cytometry. **(C)** The forming colony was verified in BV-2 cells transfected with sh-lncRNA11496 or oe-lncRNA11496. All values are presented as mean ± SD for three independent experiments (***P < 0.001, student’s *t*-test). **(D)** Cell apoptosis of BV-2 cells transfected with control plasmid, sh-lncRNA11496 and oe-lncRNA11496 was analyzed by Tunel assay. Cell apoptosis was detected using TUNEL assay kit. DAPI (blue) was used for counterstaining. The coverslips were observed and images are collected by Fluorescent Microscopy. Apoptosis cells labeled with FITC show green fluorescence.

CCK-8 assay revealed that cell proliferation was inhibited in sh-lncRNA11496 transfected BV-2 cells, while cell proliferation was accelerated in oe-lncRNA11496 transfected BV-2 cells 60 h after transfection (Figure 6A). We further verified the effect of lncRNA-11496 on the proliferation of BV-2 cells by colony formation assay. To understand the effect of lncRNA-11496 on cell cycle, BV-2 cells transfected with sh-lncRNA11496 and oe-lncRNA11496 were further subjected to cell cycle analysis (Figure 6B). Data suggested that, the number of BV-2 cells colonies was decreased when lncRNA-11496 was knocked down, while it was increased when lncRNA-11496 was overexpressed (Figure 6C). In comparison to the uninfected BV-2 cells, the proportion of cells in the S phase was significantly higher in the lncRNA-11496 knocked down (47.93% vs. 40.55%), but much less in lncRNA11496 overexpressed BV-2 cells (37.69% vs. 40.55%). Thus, the Flow cytometry assay indicated that lncRNA-11496 can affect the proliferation of BV-2 cells by inhibiting the S phase of the cells.

Finally the effect of lncRNA-11496 on apoptosis of BV-2 cells was evaluated by the TUNEL assay. The results showed that the apoptosis of BV-2 cells enhanced if lncRNA-11496 was has interfered. On the contrary the apoptosis of BV-2 cells decreased if lncRNA-11496 was overexpressed (Figure 6D). This indicates that the down-regulation of lncRNA-11496 could cause apoptosis of microglia. When BV-2 cells infected with *T. gondii*, the down-regulation of lncRNA-11496 could induce the apoptosis of the cell. We further proved that overexpression of lncRNA-11496 could rescue apoptosis in *T. gondii* infected BV-2 cells (Supplementary Figures S3A,B).

### lncRNA-11496 Regulates the Expression of MEF2C by Interaction With HDAC2

**Figure 7:**
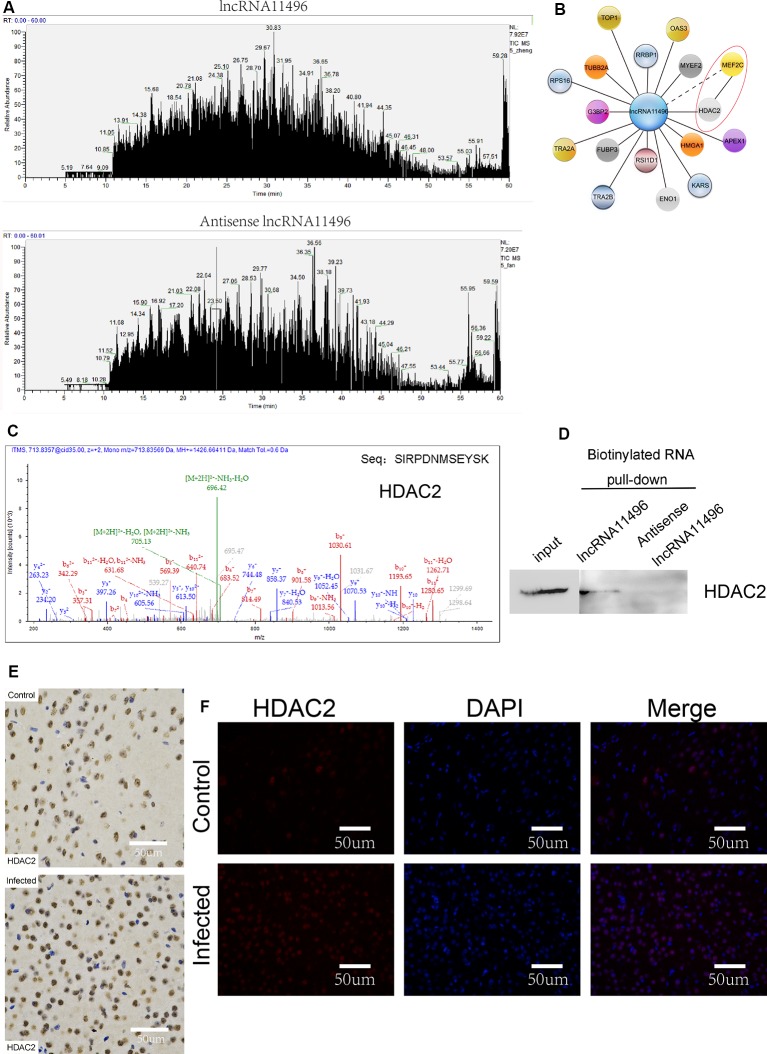
lncRNA11496 regulates the expression of MEF2C by binding to HDAC2 in chronic *T. gondii* infected mouse brain.** (A)** Mass spectrometry shows the proteins that are pulled down by lncRNA11496 and its antisense. **(B)** STRING analysis shows MEF2C could interact with HDAC2. **(C)**Mass spectrometry identified HDAC2 could bind to lncRNA11496. **(D)** The protein that was pulled down by lncRNA11496 was confirmed to be HDAC2. by Western blot. **(E)** Immunohistochemical analysis of HDAC2 expression in the brains of mice infected or uninfected with *T. gondii*. The slice derived from brain was subsequently subjected to rabbit anti- HDAC2 antibody and HRP Goat Anti-Rabbit IgG, and then nucleus stained with hematoxylin staining solution. HDAC2 protein shows in yellow color. **(F)** Immunofluorescence analysis of HDAC2 expression in the brains of mice infected or uninfected with *T. gondii*. The slice derived from brain was subjected to immunofluorescence with the primary antibody against HDAC2 (red). DAPI (blue) was used for counterstaining.

To further investigate the proteins that interact with lncRNA-11496, RNA pulldown was used to determine the proteins that interact with lncRNA-11496. The proteins interacting with lncRNA-11496 were further analyzed by Mass Spectrometry and Western blot. Mass Spectrometry showed proteins that bind to lncRNA-11496 and its antisense strand. There are about 60 proteins spectrum bind to lncRNA-11496 or its antisense strand (Figure 7A). The proteins that specifically bind to lncRNA11496 were screened by the STRING database^2^ (Figure 7B). As a result, we identified that the histone deacetylase2 (HDAC2) protein could interact with lncRNA-11496. MS analysis revealed that lncRNA11496 specifically bound to HDAC2 protein which was confirmed by Western blot analysis (Figures 7C,D).

The expression of HDAC2 protein in the brain of mice chronically infected with *T. gondii* was further detected by immunofluorescence and immunohistochemistry (Figures 7E,F). We found that the expression of HDAC2 protein was enhanced in *T. gondii* infected mouse brain compared to that of the uninfected mice. By fluorescence co-localization analysis, we showed that the MEF2C and HDAC2 were co-located in the nucleus of the cells in the brain (Supplementary Figure S4). Therefore, we suggest that lncRNA-11496 affects the expression of HDAC2 by regulating the expression of MEF2C which further influences the biological processes of nerve cells.

## Discussion

To explore the function and mechanism of lncRNA-11496 on nerve cells, BV-2 cells (a type of microglia) were used to study the function of lncRNA-11496. Microglia is an important cell line in the central nervous system and one of the major infected nerve cells (Abraham et al., 2012; Zhang et al., 2014). Chronic infection with *T. gondii* can cause microglia activation which can secrete anti-inflammatory cytokines and neurotrophic factors with potential neuroprotective properties, but excessively activated microglia could lead to neuronal apoptosis (Mahmoudvand et al., 2016; Estato et al., 2018).

In this study, lncRNAs and mRNAs’ integration expression chip (Affymetrix HTA 2.0) was performed in the brain of mice infected with the dominant *T. gondii* genotype type Chinese1 wh6 strain. We uncovered significant differential lncRNA and mRNA expression profiles between *T. gondii* infected and uninfected mice groups. Importantly, we identified a significant downregulation of lncRNA NONMMUGO11496 (lncRNA-11496) in the *T. gondii* infected mouse brain. By further validation, we confirmed that the dysregulation of lncRNA-11496 in the nucleus of cells affected cell proliferation, differentiation and apoptosis by targeting Mef2c through the potential binding to HDAC2.

It is noteworthy that lncRNA acts in different mechanisms based on its cellular location (Yang et al., 2019). For example, when lncRNA is located in the nucleus, it works by chromatin regulation or transcriptional regulation (Atianand et al., 2016). On the other hand, if it is located in the cytoplasm, it works through post-transcriptional regulation (Gong and Maquat, 2011; Fan et al., 2019). We are very interested in how lncRNA-11496 regulates the transcription of its target gene. Through cellular fraction assays, we found that the expression of lncRNA-11496 was more in the nucleus of BV-2 cells than that in the cytoplasmic fraction, demonstrating that lncRNA-11496 is mainly localized in the nucleus of BV-2 cells.

The effect of lncRNA-11496 on the proliferation, differentiation and apoptosis of the BV-2 cells was tested. We proved that overexpression of lncRNA-11496 promoted the proliferation, differentiation and apoptosis of BV-2 cells, while knocking down of lncRNA11496 inhibited the proliferation of BV-2 cells. We also found that lncRNA-11496 affects microglia proliferation by affecting the S phase of the cell cycle. S phase is a stage in which DNA synthesis occurs, the enzymes required for DNA replication are synthesized during this period (Malumbres and Barbacid, 2009). In our study, lncRNA-11496 could inhibit the DNA synthesis thus affect cell proliferation and differentiation. In our research, we also proved that the deletion of lncRNA-11496 can induce the apoptosis of microglia. It is well known that lncRNAs could play a role in apoptosis in different cell systems. For instance, it has been reported that lncRNA BANCR enhances the level of apoptosis in NSCLC (non-small cell lung carcinoma) cell lines *in vivo* by modulating the expression of Bcl-2 and BAX (Yang and Liu, 2019). Gu et al. (2019) reported that lncRNA001089 affects the proliferation, metastasis and apoptosis of glioma cells. Wan et al. (2019) suggested that LINC01125 inhibits the proliferation of breast cancer cells and affects cell apoptosis. Our study further confirmed the important role of lncRNA in apoptosis, however, providing another layer of novelty, i.e., pointing out a critical role of lncRNA-11496 in *T. gondii* infected mouse brain.

LncRNA has been shown to function as a master regulator for target gene expression. The identification of the target genes is very important to uncover the mechanism of how lncRNA function. LncRNA-mediated gene expression might get through a different mechanism, such as regulation of transcription, translation or protein modification (Ulitsky and Bartel, 2013). In this research, a target gene of lncRNA-11496 is predicted and identified as Mef2c, a member of the MEF2 subfamily, which is a transcriptional activator that can specifically bind to MEF2 elements and play a key role in neurogenesis and synaptic formation (Andzelm et al., 2019).

Mef2c has been identified in human genetic analysis as a susceptibility gene associated with many neurological diseases and is one of the biomarker genes for Alzheimer’s disease (Kamath and Chen, 2019). Mef2c is highly expressed in the brain regions associated with learning and memory, for example; the frontal cortex, the entorhinal cortex, the dentate gyrus and the amygdale (Zweier and Rauch, 2012). Studies have reported that the lack of Mef2c, or mutations within these genes are associated with neurodevelopmental disorders (Le Meur et al., 2010). Recent studies also suggest that the Mef2c can inhibit the survival of the GABA neurons by adjusting the number of synapses, synaptic structure and synaptic plasticity, and inhibit the synaptic secretion of 1-aminobutyric acid (GABA; Mitchell et al., 2018). GABA is an important inhibitory neurotransmitter in the central nervous system of the mammal (Brooks et al., 2015). Mef2c can coordinate neuronal differentiation, survival, as well as synapse formation (Adachi et al., 2016). Deletion or mutation of Mef2c can affect neuronal proliferation, differentiation and apoptosis. Synapses play an important role in regulating the mental behavior of the body. In our previous research, the apoptosis of the neurons, the decrease of the expression of the neurite and the decrease of the number of synapses were also found in the brain of mice with a chronic infection of *T. gondii* (Wang et al., 2019). These findings implied that lncRNA-11496 might be able to adjust the neurobehavior by influencing the formation of the neuro synapse by targeting Mef2c.

To further explore the mechanism of how lncRNA-11496 adjust Mef2c, we performed RNA pulldown and Mass Spectrometry (MS) analysis to find proteins that can interact with lncRNA-11496. MS and WB analysis revealed that lncRNA-11496 specifically binds to the HDAC2 protein. HDAC2 belongs to the family of histone deacetylases, which is a chromatin-modifying enzyme and an epigenetic modified regulatory transcriptional gene (Peng et al., 2015; Dou et al., 2016). Histone-modifying enzymes are crucial for modulating cell chromatin structure and gene expression in eukaryotic organisms. The imbalance of HDAC2 is associated with diseases such as schizophrenia, affective disorder, and depression. It has been reported that HDAC2 expression is significantly up-regulated in patients with depression (Schroeder et al., 2013). Immunohistochemistry and immunofluorescence assay of mice infected with *T. gondii* also revealed that the expression of HDAC2 proteins in the brain was increased. In general, increased acetylation by histone acetyltransferase protein causes an opening up of chromatin to facilitate gene transcription (Schroeder et al., 2017). STRING analysis and co-location of HDAC2 and MEF2C further implied that the HDAC2 protein can interact with the MEF2C protein and co-express in the nucleus of the cell. In this study, the expression of lncRNA-11496 was decreased after infection with *T. gondii*, which may have less binding to HDAC2, resulting in increased expression of HDAC2 in the brain.

In summary, based on the findings of lncRNA and mRNA co-expression chip for the brains of mice infected with *T. gondii* Chinese 1 Wh6 strain, we revealed that the dysregulation of lncRNA-11496 in the nucleus can affect cell proliferation, differentiation and apoptosis by targeting Mef2c which could interact with HDAC2. The neurosynaptic injury resulting from the lncRNA11496/Mef2c/HDAC2 pathway could serve as a new mechanism of mental and behavioral disorders induced by *T. gondii*. Therefore, the findings of this study provide a new experimental basis for a future novel therapeutic strategy for the treatment of this devastating disease.

## Data Availability Statement

All datasets generated for this study are included in the article/Supplementary Material.

## Ethics Statement

The animal study was reviewed and approved and all animal experimental procedures used in the present study had been given prior approval by the Institutional Animal Care and Use Committee of Shandong University under Contract LL201602044. Humane endpoints were chosen to terminate the pain or distress of experimental animals *via* euthanasia.

## Author Contributions

HC designed and supervised the study and revised the manuscript. XS performed the experiments, analyzed the data, and wrote the manuscript. TW conducted the pathology experiments on the mice. YW conducted the FISH analysis. KA extracted the RNA from the brain of the mice. GP performed the western blot analysis. YL and CZ did the bioinformatic analysis for the lncRNAs and mRNAs integration chip. SH helped to design the experiments. All authors read and approved the final version of the manuscript.

## Conflict of Interest

The authors declare that the research was conducted in the absence of any commercial or financial relationships that could be construed as a potential conflict of interest.

## Footnotes

^1^http://genome.ucsc.edu/

^2^https://string-db.org/
