# Supplementary material for: Downregulation of lncRNA-11496 in the Brain Contributes to Microglia Apoptosis via Regulation of Mef2c in Chronic T. gondii Infection Mice
Source: Front Mol Neurosci. 2020 May 15;13:77. doi: 10.3389/fnmol.2020.00077 (PMC7243434; doi:10.3389/fnmol.2020.00077)
Supplement: Supplementary file 1 [file Data_Sheet_1.pdf]

Table S1. Primer sequences used for qRT-PCR.

Primers	Forward primers	Reverse primers
lncRNA11496	ACCCAAAGTAAGCCCCGAAAT	ATAAACGAGACAATGAAGGTAGAAC
Mef2c	CTGTCTTGTCTTTGCCCTTGC	GCCATTTACGGTCCTCCTACT
GAPDH	GGACACTGAGCAAGAGAGGC	TTATGGGGGTCTGGGATGGA
U6	CAGCACATATACTAAAATTGGAACG	ACGAATTTGCGTGTCATCC

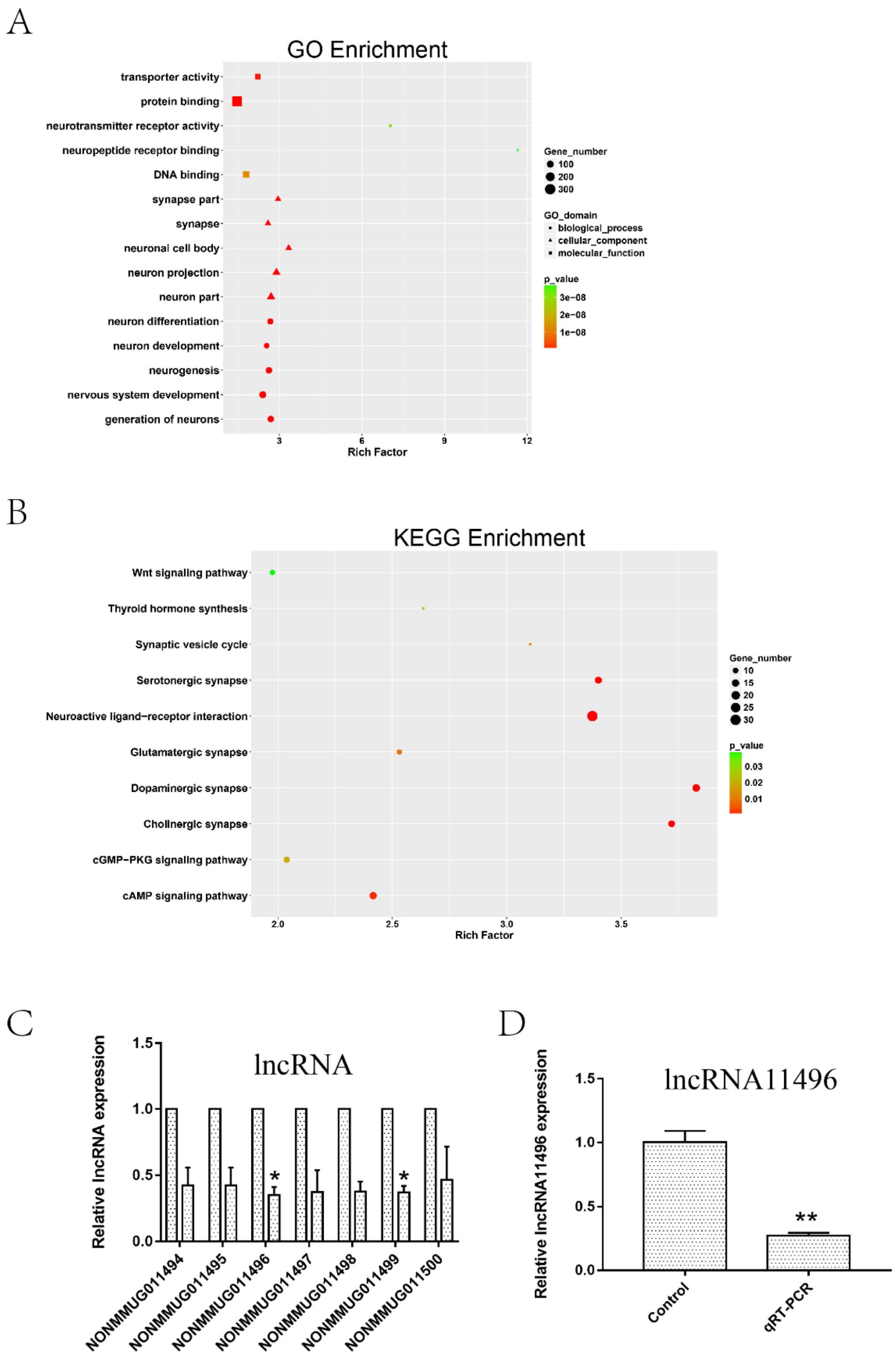

Fig.S1 KEGG and GO analysis of differentially expressed lncRNAs and mRNAs in the brains of mice infected with *T. gondii* Chinese 1 Wh6 strain and validation of the expression of lncRNA11496.

(A) GO function enrichment analysis of differentially expressed mRNAs. The x-axis indicates the subcategories, and the y-axis indicates the enrichment scores [-$\log_{10}$ (P value)] of the significant enrichment GO terms. **(B)** KEGG pathways enrichment analysis of

differentially expressed mRNAs. The vertical axis represents the pathway category and the horizontal axis represents the enrichment score [-$\log_{10}$ (P value)] of the pathway. **(C)** Significant differential expression of lncRNAs in Microarray assay. All values are presented as mean ± SD for three independent experiments. *$P < 0.05$, Student's *t*-test.

(D) Validation of lncRNA11496 expression by qRT-PCR. All values are presented as mean ± SD for three independent experiments. **$P < 0.01$, Student's *t*-test.

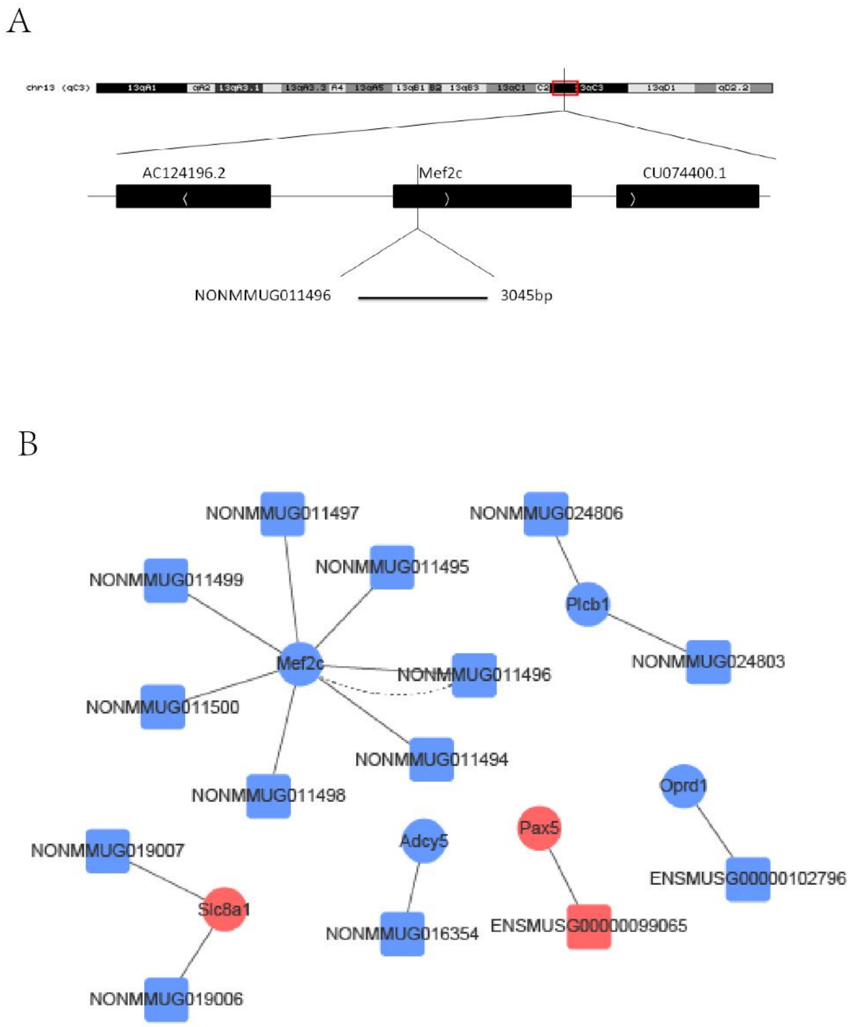

Fig.S2 Prediction of the target gene of lncRNA11496.

(A) lncRNA11496 was found to be located in the promoter region of Mef2c through the UCSC Genome Browser.

(B) The target gene of lncRNA11496 was predicted as Mef2c by cis/trans analysis.

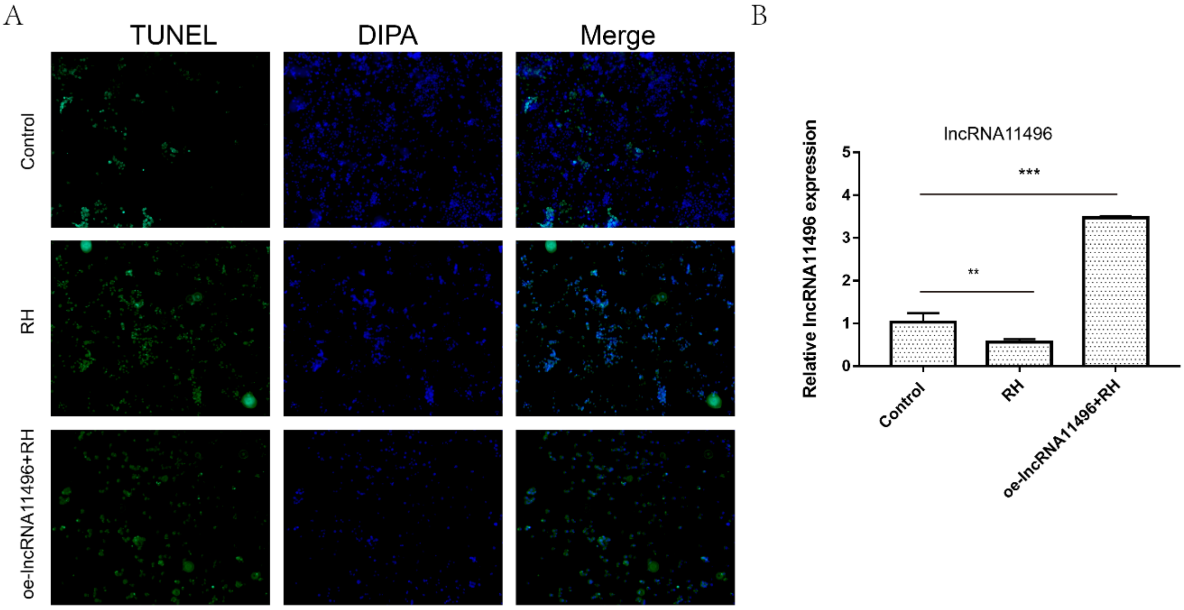

Fig.S3 Overexpression of lncRNA11496 in the BV-2 cells transfected with *T. gondii*.

(**A**) Cell apoptosis of BV-2 cell infected with *T. gondii* transfected with oe-lncRNA11496 was analyzed by TUNEL assay. The apoptosis of the cells could be rescued when overexpression of the lncRNA11496.

(**B**) Relative expression of lncRNA11496 in uninfected BV-2 cells, infected BV-2 cells and infected BV-2 cells transfected with oe-lncRNA11496 detected by qRT-PCR.

All values are presented as mean ± SD for three independent experiments. $^{**}P < 0.01$, $^{***}P < 0.001$, Student's *t*-test.

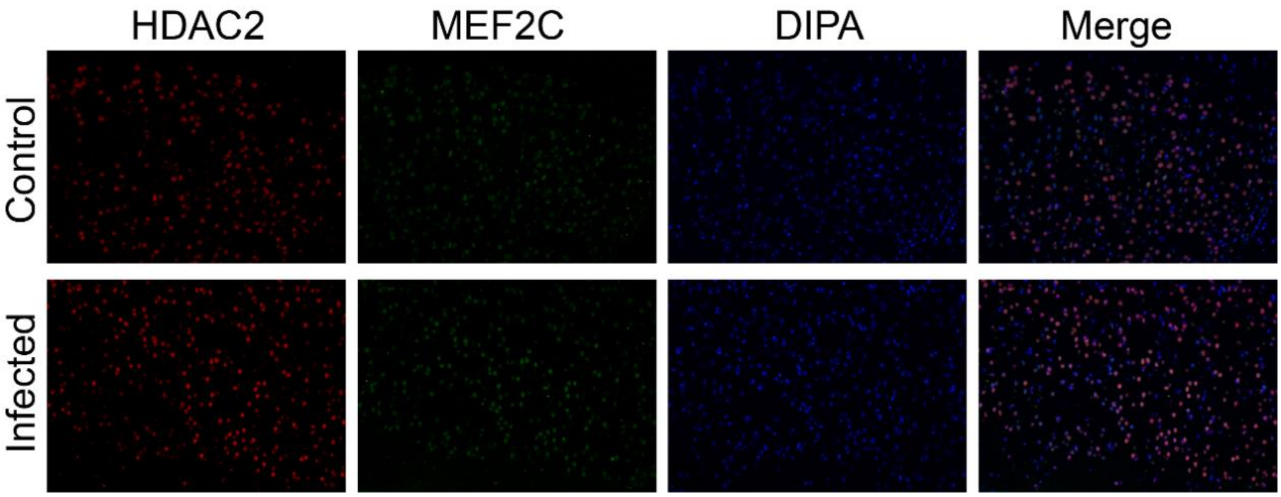

Fig.S4 Fluorescence co-localization analysis of MEF2C and HDAC2 in the *T. gondii* infected mouse brain. HDAC2 (red) and MEF2C (green) were co-located in the nucleus of the cells in the brain by fluorescence co-localization analysis.
